# Editorial: Evidence and emerging option in diagnosis and management of upper tract urothelial carcinomas

**DOI:** 10.3389/fonc.2022.1038356

**Published:** 2022-09-21

**Authors:** Liangren Liu, Yige Bao, Chengfei Liu

**Affiliations:** ^1^ Department of Urology, West China Hospital, Sichuan University, Chengdu, China; ^2^ Department of Urologic Surgery, University of California Davis, Sacramento, CA, United States; ^3^ University of California (UC) Davis Comprehensive Cancer Center, Sacramento, CA, United States

**Keywords:** upper tract urothelial carcinomas (UTUC), diagnosis, management - healthcare, biomarkers, survival data analysis

Upper tract urothelial carcinoma (UTUC) is a neglected cancer in urology (1, 2). Due to the relative rarity of UTUC, a great amount of decision-making in UTUC therapeutics comes from evidence based on bladder cancer. However, many discoveries have proven that UTUC has different features compared to bladder cancer (3). Computed tomography urography (CTU), cytology, and ureteroscopy in suspected UTUC show that nearly 60% of UTUC are invasive at the time of diagnosis, and nearly 25% are metastatic (4). There is a general lack of high-level evidence on the early diagnosis and management of UTUC, such as kidney-sparing surgery (KSS), lymph node dissection, neoadjuvant and adjuvant chemotherapy, immune checkpoint inhibition, and systemic therapy for metastatic UTUC. This Research Topic aims at recent advances in the diagnosis and therapy of UTUC, especially new techniques for early diagnosis of UTUC, KSS, lymph node dissection, neoadjuvant and adjuvant chemotherapy, and immune checkpoint inhibition in UTUC.

This is an editorial update in the field of UTUC. The 12 papers published in Frontiers in Oncology-Genitourinary Oncology by internationally renowned researchers cover five major topics:1. New diagnostic techniques for UTUC include confocal laser microscopy and optical coherence tomography (OCT). 2. Radiography techniques and biomarkers for the identification of low-risk UTUC VS aggressive UTUC. 3. Survival data for KSS and lymph node dissection in UTUC. 4. Neoadjuvant and adjuvant chemotherapy and systemic immunotherapies targeting immune checkpoint inhibition in UTUC. 5. Systemic therapy for metastatic UTUC.

The first paper reported by Fan et al. investigated the relationship between preoperative urine cytology and intravesical recurrence (IVR) in patients with UTUC in in northeast China. They performed a multicenter retrospective cohort study and demonstrated that preoperative positive urine cytology correlated with poor intravesical recurrence-free survival and can serve as a significant independent predictor of IVR. They concluded that preoperative urine cytology is a potential predictor of intravesical recurrence in patients with UTUC after radical nephroureterectomy (RNU) (5). The second paper reported by Chung et al. characterized 1095 patients with UTUC who underwent radical nephroureterectomy with bladder cuff excision (RNUx) and determined the factors affecting IVR. They found that active IVR assessment was required until 36 months after RNU. Regular screening tests, such as urine analysis and cytology, are required for patients with IVRF for >36 months. The third paper is a survey results reported by Wang et al. In this study, they conducted an online survey to analyze the knowledge and compliance of Chinese urologists with the guidelines for non-muscle-invasive bladder cancer (NMIBC) and to identify associated factors. They found that most urologists acknowledged the positive effects of these guidelines. However, compliance with some recommendations of the NMIBC guidelines remains inadequate. The fourth paper published by Chen et al. screened the TCGA dataset to identify N6-methyladenosine (m6A)- related long non-coding RNAs (lncRNAs) in bladder cancer. They constructed an m6A-related lncRNA prognostic signature (m6A-LPS) and found that it was correlated with the immune score and PD-L1 expression. In addition, m6A-LPS may play an important role in regulating tumor microenvironment. The fifth paper published by Xu et al. summarized 2561 cases of UTUC in the last 20 years in China. They found that the clinicopathological diagnostic features of UTUC in the Chinese population have changed significantly over the past 20 years, particularly in terms of patient age, sex, primary site, and multifocality. They found a significant decrease in the incidence of renal pelvic tumors, muscle invasion, and multifocal UTUC in the last 10 years, but the histological grading of the tumors remained unchanged. The sixth paper reported by Guan et al. investigated 108 patients with UTUC and performed universal immunohistochemical staining and whole-exon sequencing to detect the expression of mismatch repair (MMR) proteins and germline mutations. They found that approximately 11% of UTUC cases were suspected to have Lynch syndrome (LS) and 1.4% of cases of LS-related UTUC. The seventh paper published by Huang et al. determined the safety and feasibility of extraperitoneal laparoscopic extended lymph node dissection (LND) in UTUC patients. This prospective study suggests that the procedure provides minimal invasion, rapid recovery, and a lower risk of regional lymph node recurrence. The eighth paper published by Lee et al. further compared the benefits of LND in patients with UTUC without clinical lymph node metastasis during radical nephroureterectomy. They found no significant survival benefits related to LND in these patients. The ninth paper by Dai et al. evaluated the prognostic value of metabolic syndrome (MetS) in patients with UTUC. Their data suggested that MetS was not correlated with survival outcomes in UTUC patients. However, it was correlated with age, history of coronary heart disease, high Charlson comorbidity index, low estimated glomerular filtration rate, and low aspartate/alanine aminotransferase ratio. The tenth paper published by Zhu et al. retrospectively investigated 155 patients diagnosed with bladder cancer following RNU and explored the predictors of unfavorable pathological types of IVR following RNU. They found that operation interval, UTUC T-stage, UTUC grade, surgical approach, and hydronephrosis were independent predictors of unfavorable pathological types of IVR following RNU. The eleventh paper published by Lo et al. reported the advantage of adjuvant chemotherapy for UTUC. Their data suggested that patients who received adjuvant chemotherapy demonstrated significant survival benefits in terms of cancer-specific and disease-free survival. Their findings are consistent with the recent phase 3 trial that adjuvant platinum-based chemotherapy should be considered a new standard of care after nephroureterectomy for patient with UTUC (6). The last paper reported by Chen et al. retrospectively reviewed 302 patients with UTUC who underwent RNU with bladder cuff excision. They found that the tumor location was an independent predictor of local recurrence. Ureter tumors may be associated with worse oncological outcomes, especially local recurrence of UTUC.

In summary, the papers included in this Research Topic provide an emerging update and new avenue on UTUC. We would like to express our sincere gratitude to all authors, editors, and reviewers of these publications, as well as the editorial team at Frontiers for their devotion and assistance in the process of reviewing and publishing this Research Topic.

## Author contributions

CL wrote the editorial. LL and YB edited the editorial. All authors contributed to the article and approved the submitted version.

## Acknowledgments

We would like to express our sincere gratitude to all authors, editors, and reviewers of these publications, as well as the editorial team at Frontiers for their devotion and assistance in the process of reviewing and publishing this Research Topic.

## Conflict of interest

The authors declare that the research was conducted in the absence of any commercial or financial relationships that could be construed as a potential conflict of interest.

## Publisher’s note

All claims expressed in this article are solely those of the authors and do not necessarily represent those of their affiliated organizations, or those of the publisher, the editors and the reviewers. Any product that may be evaluated in this article, or claim that may be made by its manufacturer, is not guaranteed or endorsed by the publisher.
